# Usefulness of short-term eltrombopag treatment as a supportive treatment in hepatocellular carcinoma patients with cirrhosis and severe thrombocytopenia: A report of two cases

**DOI:** 10.3892/ol.2014.1976

**Published:** 2014-03-14

**Authors:** TAKUMI KAWAGUCHI, MASAHITO NAKANO, MANABU SATANI, SHUJI SUMIE, SHINGO YAMADA, KEISUKE AMANO, RYOKO KUROMATSU, MICHIO SATA

**Affiliations:** 1 Division of Gastroenterology, Department of Medicine, Kurume University School of Medicine, Kurume, Fukuoka 830-0011, Japan; 2 Department of Digestive Disease Information and Research, Kurume University School of Medicine, Kurume, Fukuoka 830-0011, Japan

**Keywords:** chronic liver disease, thrombopoietin receptor, hepatoma, growth, megakaryocyte growth and development factor

## Abstract

Eltrombopag is an oral thrombopoietin (TPO) receptor agonist that increases platelet counts in patients with idiopathic thrombocytopenic purpura and in patients with liver cirrhosis. When cirrhotic patients with thrombocytopenia undergo elective invasive procedures, eltrombopag treatment reduces the requirement for platelet transfusions. However, TPO is known to have proliferative effects on hepatic progenitor cells and hepatic sinusoidal endothelial cells, which indicates that eltrombopag may accelerate tumor progression. Thus, the effect of eltrombopag on hepatocellular carcinoma (HCC) progression is an important issue. The current study describes two cases of HCC with cirrhosis-related thrombocytopenia. A two-week administration of eltrombopag increased platelet counts from 4.8 to 11.3×10^4^ /μl in case 1 and 4.5 to 23.2×10^4^ /μl in case 2. However, no changes were identified in the serum levels of tumor markers or HCC size following eltrombopag administration in the two cases. These HCCs were curatively treated by radiofrequency ablation without platelet transfusions or serious bleeding. Thus, short-term eltrombopag administration may not accelerate HCC proliferation and may be beneficial for invasive HCC treatment in cirrhotic patients with thrombocytopenia.

## Introduction

Thrombocytopenia is a common complication of liver cirrhosis and is an intractable issue when performing invasive procedures (1,2). When cirrhotic patients with severe thrombocytopenia undergo elective invasive procedures for hepatocellular carcinoma (HCC), ~60% of the patients require treatment for thrombocytopenia, including platelet transfusions, partial splenic embolization or splenectomy (2). However, platelet transfusions carry the risk of anaphylactic shock, infection and transfusion-related acute lung injuries. Partial splenic embolization may lead to splenic abscesses. In addition, splenectomy may result in splenic vein thrombosis and overwhelming post-splenectomy infections. Thus, a non-invasive therapeutic strategy for thrombocytopenia is required for cirrhotic patients with HCC.

Eltrombopag is an oral thrombopoietin (TPO)-receptor (R) agonist that stimulates megakaryocytes and thus, increases platelet counts in patients with idiopathic thrombocytopenic purpura (3). Since decreased plasma levels of TPO, in addition to hypersplenism, are major pathogenic features of cirrhosis-related thrombocytopenia (1), eltrombopag appears to improve thrombocytopenia in cirrhotic patients. Furthermore, eltrombopag has previously been reported to increase platelet counts and thus, enables subsequent interferon treatments and invasive procedures in cirrhotic patients with severe thrombocytopenia (4–6).

TPO is known as a megakaryocyte growth and development factor that leads to megakaryocyte proliferation. In addition, TPO induces the proliferation of various cell types that express the TPO-R. In the liver, the TPO-R occurs in hepatic progenitor cells (7) and hepatic sinusoidal endothelial cells (8). In addition, TPO induces the proliferation of these two cell types (7,8). Since hepatic sinusoidal endothelial cells and hepatic progenitor cells are crucial in the development and progression of HCC, the effect of eltrombopag on the proliferation of HCC is significant.

The current report presents two cases of HCC with cirrhosis-related thrombocytopenia. In these two cases, eltrombopag treatment increased platelet counts without any concomitant changes in the serum levels of tumor markers or HCC size. The HCCs were subsequently treated by curative radiofrequency ablation (RFA) without platelet transfusions or serious bleeding. Patient provided writted informed consent.

## Case report

### Case 1

In 2008, a 65-year-old male patient was referred to the Kurume University Hospital (Kurume, Japan) for the treatment of hepatitis C virus (HCV)-related liver cirrhosis. The patient had a history of esophageal varices rupture (that occurred in 1993) and HCC, which was treated by transcatheter hepatic arterial chemolipiodolization with embolization in 2007.

**Figure 1:**
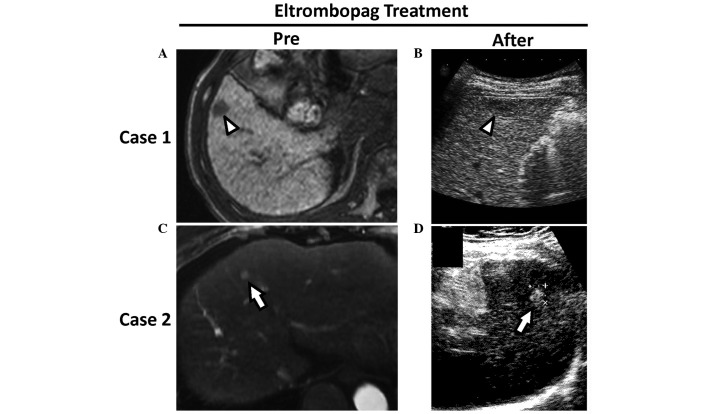
Effect of eltrombopag on HCC size. (A) MRI scan showed a space-occupying lesion with a maximum diameter of ~15 mm in Couinaud segment 5 of the liver. The lesion showed decreased contrast uptake in the hepatocyte phase following injection of EOB. (B) Two weeks following the eltrombopag treatment, ultrasonography showed a hypoechoic lesion with a maximum diameter of ~15 mm in Couinaud segment 5 of the liver. (C) MRI scan showed a space-occupying lesion with a maximum diameter of ~7 mm in Couinaud segment 4 of the liver. The lesion showed contrast enhancement in the early phase following injection of EOB. (D) Two weeks following the eltrombopag treatment, ultrasonography showed a hyperechoic lesion with a maximum diameter of ~8 mm in Couinaud segment 4 of the liver. HCC, hepatocellular carcinoma; MRI, magnetic resonance imaging; EOB, gadolinium-ethoxybenzyl-diethylenetriamine pentaacetic acid.

The patient was treated with ursodeoxycholic acid, glycyrrhizin and nutritional therapy, including branched-chain amino acid granules. However, in 2009, a regular abdominal ultrasound revealed a hypoechoic lesion with a maximum diameter of ~15 mm in Couinaud segment 5 of the liver. The lesion showed decreased contrast uptake in the hepatocyte phase as determined by gadolinium-ethoxybenzyl-diethylenetriamine pentaacetic acid (EOB)-enhanced magnetic resonance imaging (MRI) scan (Fig. 1A). Additionally, an aspiration tumor biopsy revealed that the lesion was a well-differentiated HCC. The HCC was a single nodule and the patient’s Child-Pugh score was 7 points. Although RFA was selected as a therapeutic strategy for HCC, the patient’s platelet count (4.8×10^4^ /μl) revealed severe thrombocytopenia.

**Figure 2:**
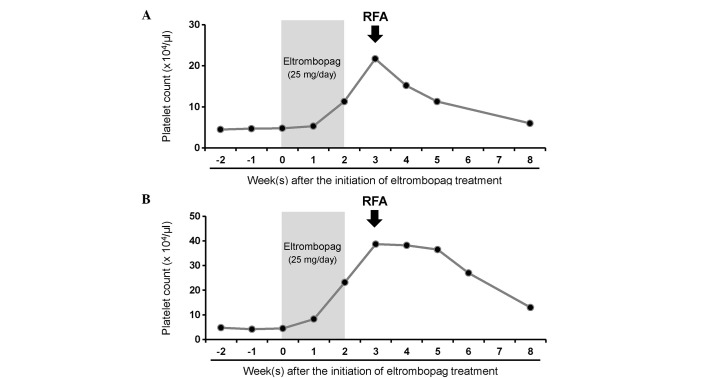
Changes in platelet count following the initiation of eltrombopag treatment. (A) In case 1, the platelet count increased from 4.8 to 11.3×10^4^ /μl during eltrombopag treatment (gray area). Following the termination of eltrombopag treatment, the platelet count increased to 21.7×10^4^ /μl (three weeks since the initiation of eltrombopag treatment). (B) In case 2, the platelet count increased from 4.5 to 23.2×10^4^ /μl during eltrombopag treatment (gray area). Following the termination of eltrombopag treatment, the platelet count increased further to 38.7 ×10^4^ /μl (three weeks since the initiation of eltrombopag treatment).

The risks and benefits of participation in the phase II study were explained to the patient (5); this study aimed to investigate the efficacy and safety of eltrombopag in patients with thrombocytopenia. Written informed consent was obtained and 25.0 mg eltrombopag was subsequently administered to the patient. The patient’s platelet count increased from 4.8 to 11.3×10^4^ /μl during the administration of eltrombopag (Fig. 2A; gray area). Following the termination of eltrombopag treatment, the platelet count continued to increase to 21.7×10^4^ /μl at three weeks since the initiation of eltrombopag treatment. No significant changes were observed in the serum levels of α-fetoprotein and des-γ-carboxy prothrombin (Table I) or HCC size (Fig. 1B). The HCC was curatively treated by RFA without platelet transfusion or serious bleeding at three weeks following initiation of the eltrombopag treatment.

### Case 2

In 2005, a 61-year-old female patient was referred to the Kurume University Hospital for the treatment of HCV-related liver cirrhosis. The patient was treated with ursodeoxycholic acid and glycyrrhizin. However, the hepatic fibrosis gradually progressed. In 2009, a regular abdominal ultrasound revealed a hyperechoic lesion with a maximum diameter of ~7 mm in Couinaud segment 4 of the liver. An EOB-MRI scan revealed contrast enhancement of the lesion during the early phase (Fig. 1C). The lesion was subsequently diagnosed as a well-differentiated HCC via an aspiration tumor biopsy. The HCC was a single nodule and the patient’s Child-Pugh score was 8 points. RFA was selected as a therapeutic strategy for the HCC, however, the patient’s platelet count decreased to 4.5×10^4^ /μl.

The risks and benefits of participation in the phase II study were explained as previously described (5). Written informed consent was obtained and 25.0 mg eltrombopag was subsequently administered to the patient. The platelet count increased from 4.5 to 23.2×10^4^ /μl during eltrombopag treatment (Fig. 2B; gray area). Following the termination of eltrombopag treatment, the platelet count increased further to 38.7×10^4^ /μl (three weeks since the initiation of eltrombopag treatment). No significant changes were observed in the serum levels of α-fetoprotein and des-γ-carboxy prothrombin (Table I) or in the HCC size (Fig. 1B). At three weeks following the initiation of eltrombopag treatment, the HCC was curatively treated by RFA without platelet transfusion or serious bleeding.

## Discussion

The effect of eltrombopag on the progression of malignant neoplasms is an important issue. The current report presents two cases of HCC with cirrhosis-related severe thrombocytopenia. The administration of eltrombopag increased the platelet count in these two cases without inducing significant changes in the serum levels of tumor markers or HCC size. These HCCs were successfully treated by RFA without platelet transfusions or serious bleeding. The observations from these cases may indicate that the short-term administration of eltrombopag does not affect the progression of HCC and may be useful when administered prior to invasive procedures in cirrhotic patients with thrombocytopenia.

In the present study, eltrombopag treatment significantly increased platelet count, which subsequently resulted in successful RFA therapy and the avoidance of platelet transfusions. These beneficial observations are consistent with those of previous reports. Eltrombopag has previously been shown to improve thrombocytopenia and permit interferon therapy in patients with cirrhosis that is associated with HCV infection (6). More recently, eltrombopag has been reported to reduce the requirement for platelet transfusions in cirrhotic patients who have undergone elective invasive procedures (4). The study included patients with HCC, however, no monitoring of tumor markers or HCC size was performed, which was conducted in the present study. Thus, eltrombopag may replace platelet transfusions, partial splenic embolization or splenectomy when elective invasive procedures are performed on cirrhotic patients with thrombocytopenia.

In the current two cases, thrombosis did not develop during or following eltrombopag treatment. However, certain previous clinical trials have reported that invasive procedures increase the risk for the development of thrombosis, even in cirrhotic patients with thrombocytopenia (4,5). The risk is proportional to the increasing platelet counts of >200,000/mm^3^, which was avoided in the present study by close monitoring of the patients. Although the reason for the development of thrombosis remains unclear, von Willebrand factor levels have been found to increase as an adaptive mechanism in response to reduced platelet adhesion abilities in cirrhotic patients (9). Additionally, an increase was identified in the platelet formation of isoprostanes, which promote platelet activation via the upregulation of proaggregatory factors in cirrhotic patients (10,11). Alternatively, impaired liver function may be involved in the development of thrombosis, since the majority of eltrombopag is eliminated in the liver (12). Higher plasma concentrations of eltrombopag were observed in patients with Child-Pugh class B when compared with patients with Child-Pugh class A (5). Thus, patients must be vigilant for signs of thrombosis, in particular those with advanced liver cirrhosis, during eltrombopag treatment.

Hepatic progenitor cells and hepatic sinusoidal endothelial cells express TPO-R, in addition TPO induces the proliferation of these two types of cells (7,8). Furthermore, TPO-R is expressed in hepatoma cell lines, such as Huh7, Hep3B and HepG2 (13,14); these observations indicate that eltrombopag may accelerate the progression of HCC. Although eltrombopag has been administered to treat tumor-bearing patients (4,15), the effect of eltrombopag on tumor progression has not previously been reported. In the present study, it was demonstrated that short-term eltrombopag treatment did not accelerate the progression of HCC. Although, from the results of the two cases the possibility that long-term administration of eltrombopag may accelerate tumor progression cannot be dismissed; however, subsequent findings may support the hypothesis that short-term administration of eltrombopag does not accelerate the proliferation of HCC.

TPO-R expression has previously been found to be lower in hepatoma cell lines compared with expression in primary hepatocytes (14) and HCC tissues do not express TPO-R (13). In addition, TPO does not activate the extracellular signal-regulated kinases 1/2 or the signal transducers and activators of transcription 3 and 5 pathways, or affect the proliferation, migration or invasion of Huh7 cells (14). Furthermore, TPO does not upregulate Bax, Bcl-2 or cleaved caspase 3 and does not induce anti-apoptotic effects in Huh7 cells (14). Finally, in a previous xenograft experiment, no significant differences were identified in the tumor volume, tumor appearance and histological morphology of Huh7 cells that were treated with or without TPO (14). Thus, the short-term administration of eltrombopag may not accelerate tumor progression.

In conclusion, the present study described two cases of HCC with cirrhosis-related thrombocytopenia. Eltrombopag treatment increased the platelet count in the two cases and resulted in successful RFA treatments without platelet transfusions or serious bleeding. Additionally, no changes were observed in the serum levels of tumor markers or HCC size. Although eltrombopag is not currently used for the treatment of thrombocytopenia in patients with chronic liver disease undergoing invasive procedures, the observations of the present study indicated that the short-term administration of eltrombopag may not accelerate the progression of HCC and may be beneficial for HCC treatment in cirrhotic patients with thrombocytopenia.

## Figures and Tables

**Table I:** Changes in biochemical parameters during the course of eltrombopag treatment.

		Following eltrombopag initiation					
		Case 1			Case 2		
			
Biochemical parameter	Reference value	Pre	Week 2	Week 3	Pre	Week 2	Week 3
Red blood cells, ×10^4^/mm^3^	430–570	383	368	352	394	369	358
Hemoglobin, g/dl	14.0–18.0	12.5	11.7	11.5	13.1	12.3	12.1
White blood cells, /mm^3^	4000–9000	2200	2500	2600	3800	4200	5200
Platelets, ×10^4^/mm^3^	13–36	4.8	11.3	21.7	4.5	23.2	38.7
Aspartate transaminase, U/l	13–33	102	67	60	46	35	36
Alanine aminotransferase, U/l	8–42	96	64	49	35	24	22
Lactate dehydrogenase, U/l	119–229	285	216	249	298	344	341
Alkaline phosphatase, U/l	115–359	352	372	339	278	245	301
γ-glutamyl transpeptidase, U/l	10–47	29	28	27	25	27	39
Total protein, g/dl	6.70–8.30	8.09	7.74	7.59	7.71	7.17	7.34
Albumin, g/dl	4.00–5.00	2.83	2.69	2.59	3.23	2.91	2.87
Total bilirubin, mg/dl	0.30–1.50	1.19	0.99	0.64	2.09	1.99	1.40
Prothrombin activity, %	60–130	64	59	69	70	66	73
Prothrombin activity, INR	0.87–1.25	1.32	1.40	1.27	1.25	1.29	1.22
BUN, mg/dl	8.0–22.0	22.6	21.5	25.4	15.0	15.3	20.2
Creatinine, mg/dl	0.60–1.10	1.00	0.96	1.21	0.64	0.68	0.81
Sodium, mEq/l	138–146	137	136	136	139	138	134
Child-Pugh score		7	7	7	8	8	7
MELD score		10	10	11	12	12	10
α-fetoprotein, ng/ml	<8.7	23.0	21.8	26.2	27.8	30.5	23.9
Des-γ-carboxy prothrombin, mAU/ml	<40	16	14	20	45	35	30

BUN, blood urea nitrogen; MELD, model for end-stage liver disease; INR, international normalized ratio.

## References

[1] Afdhal N, McHutchison J, Brown R (2008). Thrombocytopenia associated with chronic liver disease. J Hepatol.

[2] Kawaguchi T, Kuromatsu R, Ide T (2009). Thrombocytopenia, an important interfering factor of antiviral therapy and hepatocellular carcinoma treatment for chronic liver diseases. Kurume Med J.

[3] Bussel JB, Cheng G, Saleh MN (2007). Eltrombopag for the treatment of chronic idiopathic thrombocytopenic purpura. N Engl J Med.

[4] Afdhal NH, Giannini EG, Tayyab G, ELEVATE Study Group (2012). Eltrombopag before procedures in patients with cirrhosis and thrombocytopenia. N Engl J Med.

[5] Kawaguchi T, Komori A, Seike M (2012). Efficacy and safety of eltrombopag in Japanese patients with chronic liver disease and thrombocytopenia: a randomized, open-label, phase II study. J Gastroenterol.

[6] McHutchison JG, Dusheiko G, Shiffman ML, TPL102357 Study Group (2007). Eltrombopag for thrombocytopenia in patients with cirrhosis associated with hepatitis C. N Engl J Med.

[7] Schmelzer E, Deiwick A, Bruns H, Fiegel HC, Bader A (2008). Thrombopoietin is a growth factor for rat hepatic progenitors. Eur J Gastroenterol Hepatol.

[8] Cardier JE, Dempsey J (1998). Thrombopoietin and its receptor, c-mpl, are constitutively expressed by mouse liver endothelial cells: evidence of thrombopoietin as a growth factor for liver endothelial cells. Blood.

[9] Lisman T, Bongers TN, Adelmeijer J (2006). Elevated levels of von Willebrand Factor in cirrhosis support platelet adhesion despite reduced functional capacity. Hepatology.

[10] Basili S, Raparelli V, Riggio O, CALC Group (2011). NADPH oxidase-mediated platelet isoprostane over-production in cirrhotic patients: implication for platelet activation. Liver Int.

[11] Violi F, Pignatelli P (2012). Eltrombopag before procedures in patients with cirrhosis and thrombocytopenia. N Engl J Med.

[12] Haselboeck J, Kaider A, Pabinger I, Panzer S (2013). Function of eltrombopag-induced platelets compared to platelets from control patients with immune thrombocytopenia. Thromb Haemost.

[13] Columbyova L, Loda M, Scadden DT (1995). Thrombopoietin receptor expression in human cancer cell lines and primary tissues. Cancer Res.

[14] Nozaki R, Murata S, Nowatari T (2012). Effects of thrombopoietin on growth of hepatocellular carcinoma: Is thrombopoietin therapy for liver disease safe or not?. Hepatol Res.

[15] Chawla SP, Staddon A, Hendifar A, Messam CA, Patwardhan R, Kamel YM (2013). Results of a phase I dose escalation study of eltrombopag in patients with advanced soft tissue sarcoma receiving doxorubicin and ifosfamide. BMC Cancer.

